# Dihydroisocoumarins from *Radix* Glycyrrhizae

**DOI:** 10.1186/s13065-018-0427-0

**Published:** 2018-05-11

**Authors:** Songsong Zhao, Xinjia Yan, Ying Zhao, Jing Wen, Zhenzhen Zhao, Hongwei Liu

**Affiliations:** 1 School of Pharmacy, Harbin University of Commerce, Harbin, 150076 China; 2 Department of Educational Administration, First Affiliated Hospital of Jinzhou Medical University, Jinzhou, 121000 China; 3 Department of Head and Neck Surgery, Cancer Hospital of China Medical University, Shenyang, 110042 China

**Keywords:** *Radix* Glycyrrhizae, Isocoumarin, ECD investigation, NMR spectrum

## Abstract

**Background:**

*Radix* Glycyrrhizae is the rhizome of *Glycyrrhiza inflata* Bat., *Glycyrrhiza uralensis* Fisch. or *Glycyrrhiza glabra* L. The present paper describes the isolation and the structural elucidation of three new dihydroisocoumarins obtained from the 70% EtOH extract of *Radix* Glycyrrhizae. And the cytotoxic activities of these new compounds were also evaluated using four cell lines, subsequently.

**Results:**

A pair of new dihydroisocoumarin epimers ((3*R*,4*S*)-4,8-dihydroxy-3-methyl-1-oxoisochroman-5-yl)methyl acetate (**1**) and ((3*R*,4*R*)-4,8-dihydroxy-3-methyl-1-oxoisochroman-5-yl)methyl acetate (**2**) along with a new dihydroisocoumarin (3*R*,4*R*)-4,8-dihydroxy-3,5-dimethylisochroman-1-one (**3**) were isolated from *Radix* Glycyrrhizae. Their structures were elucidated on the basis of chemical and spectral analysis, including 1D, 2D NMR analyses, HR–ESI–MSand ECD calculation comparing with those of experimental CD spectra. Cytotoxic activities of the three compounds were evaluated using the HepG2, A549, LoVo and Hela cell lines, respectively. IC_50_ values indicated compounds **1**–**3** exhibited moderate or less cytotoxic activity in vitro.

**Conclusions:**

Dihydroisocoumarin is not the common components in *Radix* Glycyrrhizae, a series of dihydroisocoumarin were obtained in this plant could be a supplement to the chemical study of this plant.

**Electronic supplementary material:**

The online version of this article (10.1186/s13065-018-0427-0) contains supplementary material, which is available to authorized users.

## Background

**Fig. 1:**
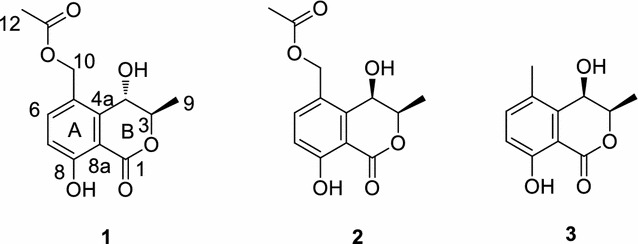
Structures of compounds **1**–**3**

*Radix* Glycyrrhizae is the rhizome of *Glycyrrhiza inflata* Bat., *Glycyrrhiza uralensis* Fisch. or *Glycyrrhiza glabra* L. They are widely distributed in the northwest and northeast of China [1]. The pharmacological activities of *Radix* Glycyrrhizae are mainly represented by the main triterpene saponins, glycyrrhizin, glycyrrhizic acid, glycyrrhizinic acid and its aglycone, glycyrrhetinic acid [2, 3]. Its root possesses wide broad pharmacological actions. According to literature reports, its pharmacological activities include the following aspects: effects on central nerve system; cardiovascular system and endocrine system; liver, renal and pancreas functions, anti-ulcer action, anticancer action, anti-allergic and anti-inflammatory effects, anti-virus and antibacteria activities, and effect on immune function and so on [4, 5]. In this paper, we describe the isolation and the structural elucidation of three new dihydroisocoumarins obtained from the 70% ethyl alcohol (EtOH) extract of *Radix* Glycyrrhizae. Their structures (Fig. 1) were established by extensive spectroscopic data analysis and comparison with those of literature values.

## Results and discussion

**Fig. 2:**
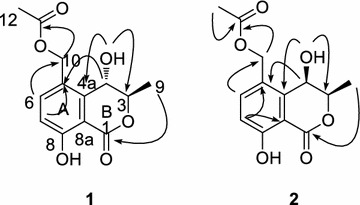
The key HMBC (→) correlations of compound **1** and **2**

Compound **1** was obtained as yellow crystal (CH_3_OH), with the molecular formula C_13_H_14_O_6_ as determined by high resolution electrospray ionization mass spectra (HR–ESI–MS) at *m/z* 289.0681 [M + Na]^+^, indicating the presence of seven degrees of unsaturation. The ^1^H-NMR spectrum of compound **1** (Table 1) displayed one hydroxyl proton signal at *δ*_H_11.20 (1H, s), two methyl signals at *δ*_H_1.45 (3H, d, *J* = 6.4 Hz) and 2.04 (3H, s), two aromatic proton signals at *δ*_H_6.99 (1H, d, *J* = 8.8 Hz) and 7.64 (1H, d, *J* = 8.8 Hz), along with some other methylene and methine proton signals [*δ*_H_5.70 (1H, d, J = 7.2 Hz), 5.09 (1H, d, J = 12.4 Hz), 5.17 (1H, d, *J* = 12.4 Hz), 4.66 (1H, dd, *J* = 6.8, 1.6 Hz) and 4.72 (1H, qd, *J* = 6.4, 1.6 Hz)]. The ^13^C-NMR spectrum of compound **1** (Table 1) showed 13 carbon signals, including eight sp^2^ carbons (*δ*_C_169.8, 124.7, 138.3, 117.3, 161.1, 170.8, 141.3 and 108.2), three oxygenated sp^3^ carbons (*δ*_C_78.5, 62.3 and 62.2), and two methyl carbons (*δ*_C_16.6 and 21.2). All the NMR data and the degree of unsaturation revealed the presence of a 1,2,3,4-substituted benzyl group and a lactone ring. Signal assignments were specified by Heteronuclear Single Quantum Coherence (HSQC) experiment. The Heteronuclear Multiple-Bond Correlation (HMBC) spectrum (Fig. 2) showed the long-rang correlations between H-7 at *δ*_H_6.99and C-8a at *δ*_C_108.2, C-5 at *δ*_C_124.7, C-8 at *δ*_C_161.1; between H-6 at *δ*_H_7.64 and C-10 at *δ*_C_62.2, C-8a at *δ*_C_108.2, C-4a at *δ*_C_141.3; between H-10 at *δ*_H_5.09/5.17 and C-5 at *δ*_C_124.7, C-4a at *δ*_C_ 141.3, C-11 at *δ*_C_ 170.8, between H-9 at *δ*_H_1.45 and C-4 at *δ*_C_62.3, between H-4 at *δ*_H_4.66 and C-3 at *δ*_C_78.5, C-4a at *δ*_C_ 141.3, C-5 at *δ*_C_ 124.7, between H-4 at *δ*_H_4.66 and C-5 at *δ*_C_124.7, which led to a conclusion that the planar structure of compound **1** was similar to that of (3*R*, 4*R*)-4,8-dihydroxy-5-(hydroxymethyl)-3-methylisochroman-1-one [6], except for the acetylated of hydroxyl groups at C-10. Thus, the planar structure of **1** was determined as shown in Fig. 1.

**Table 1:** ^1^H NMR (600 MHz) and ^13^C NMR (150 MHz) spectral data of compounds **1**–**3** in DMSO-*d*_*6*_

Position	Compound 1		Compound 2		Compound 3	
	*δ*_C_	*δ*_H_ (*J* in Hz)	*δ*_C_	*δ*_H_ (*J* in Hz)	*δ*_C_	*δ*_H_ (*J* in Hz)
1	169.8	–	167.6	–	168.0	–
2	–	–	–	–	–	–
3	78.5	4.72 (1H, qd, 6.4, 1.6)	80.7	4.88 (1H, qd, 6.8, 1.6)	80.9	4.87 (1H, qd, 6.8, 1.2)
4	62.3	4.66 (1H, dd, 6.8, 1.6)	63.2	4.77 (1H, dd, 5.2, 1.6)	63.6	4.67 (1H, dd, 5.6, 1.2)
5	124.7	–	125.3	–	127.0	–
6	138.3	7.64 (1H, d, 8.8)	138.3	7.66 (1H, d, 8.8)	137.1	7.45 (1H, d, 8.4)
7	117.3	6.99 (1H, d, 8.8)	117.0	7.03 (1H, d, 8.8)	116.7	6.92 (1H, d, 8.4)
8	161.1	–	160.6	–	158.8	–
9	16.6	1.45 (3H, d, 6.4)	17.4	1.16 (3H, d, 6.8)	16.7	1.16 (3H, d, 6.8)
10	62.2	5.09 (1H, d, 12.4)	62.0	5.07 (1H, d, 12.4)	17.6	2.29 (3H, s)
		5.17 (1H, d, 12.4)		5.20 (1H, d, 12.4)		
11	170.8	–	170.2			
12	21.2	2.04 (3H, s)	20.7	2.03 (3H, s)		
4a	141.3	–	138.9	–	138.6	
8a	108.2	–	107.4	–	107.1	
OH-4		5.70 (1H, d, 7.2)		5.96 (1H, d, 5.2)	–	5.82 (1H, d, 5.2)
OH-8		11.20 (1H, s)		11.19 (1H, s)	–	10.9 (1H, s)

Chemical shift values are expressed in ppm

**Fig. 3:**
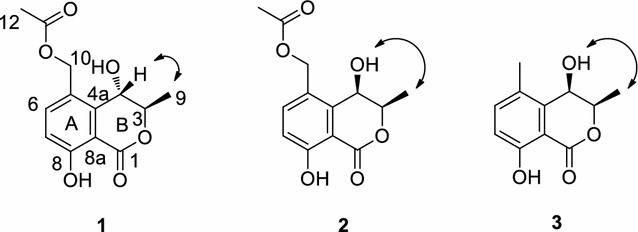
The key NOESY ( ) correlations of compound **1**–**3**

Compound **2** was also obtained as yellow crystal. The molecular formula was determined to be C_13_H_14_O_6_ by HR–ESI–MS at *m/z* 289.0670 [M + Na]^+^. The ^1^H and ^13^C NMR signals of **2** were almost identical to those of **1** with slight difference at C-1, C-3, C-4, C-5, and C-4a. The CD spectrum of **2** gave an exactly opposite absorption band at 250 nm compared with that of **1**, and thus **2** was suggested to be the epimer of **1** at C-3. HMBC correlations of **2** shown in Fig. 3 verified the planar structure of **2**, which was the same as that of **1**. The relative configurations of **1** and **2** were established by NOESY analysis (Fig. 3). For compound **2**, NOESY cross-peak between active proton of C-4 and H-3 was given while for compound **1**, NOESY cross-peak between active proton of C-4 and 9-CH_3_ was observed, indicating the axial orientation of the active proton of C-4 as C-4 active proton could only give one NOESY cross-peak with either H-3 or 9-CH_3_.

**Fig. 4:**
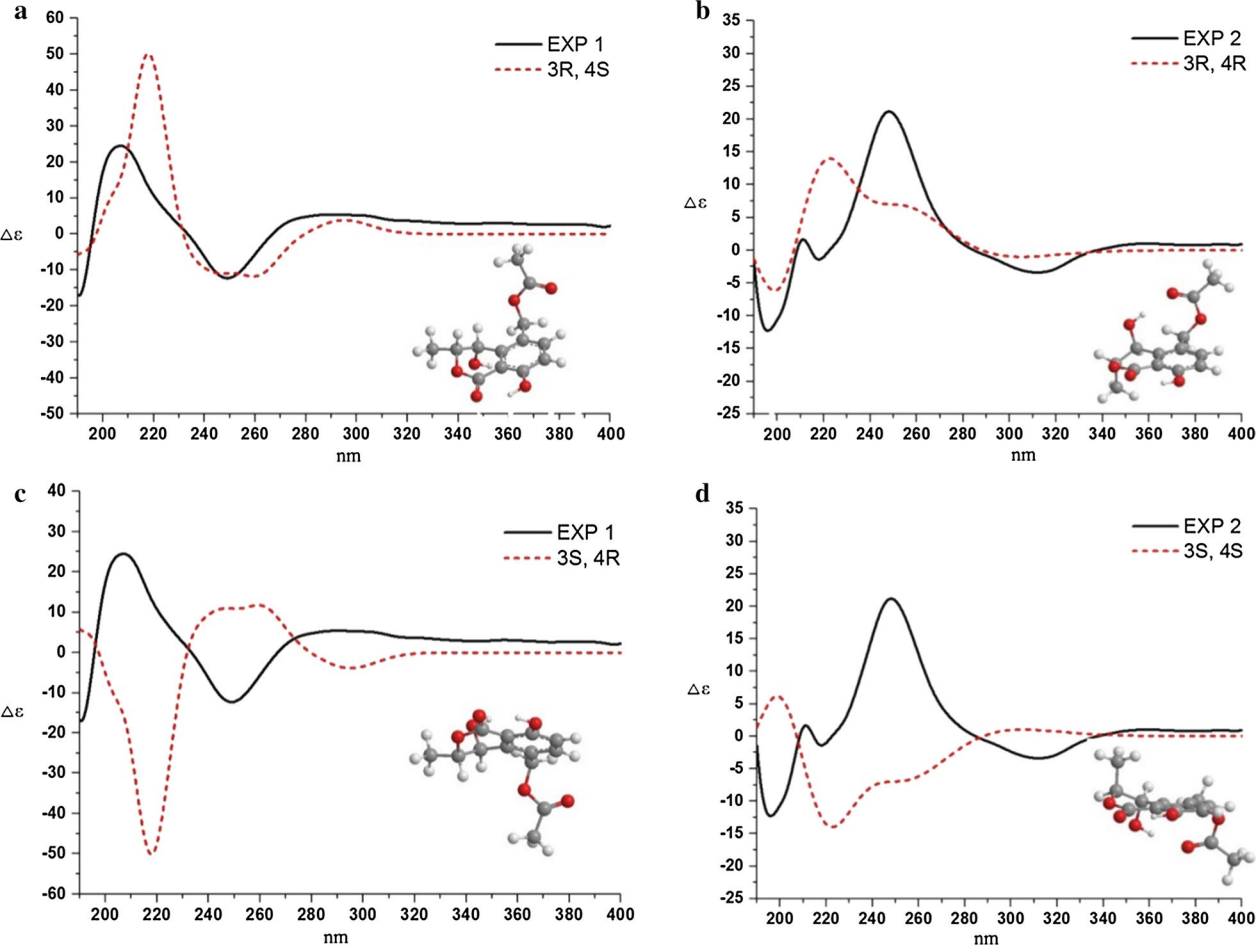
ECD results of **1** and **2**. Absolute configurations of C-3 and C-4: **a**, 3R, 4S; **b**, 3R, 4R; **c**, 3S, 4R; **d**, 3S, 4S

The ECD (Electronic Circular Dichroism Spectroscopy) calculating study of **1** and **2** was performed based on the relative configuration of **1** and **2**. Having two chiral centers, there are four possible stereo-isomers for 4,8-dihydroxy-3-methyl-1-oxoisochroman-5-yl)methyl acetate as shown in Fig. 4. The ECD results of each possible isomer and the experimental CD (Circular Dichroism Spectroscopy) curves of **1** and **2** were also expressed in Fig. 4a, b. The ECD results were represented in shot dashed line in Fig. 4a, d that both gave negative cotton effect at 250 nm, and so did Fig. 4b, c that both exhibited positive cotton effect at 250 nm, indicating that C-4 orientation dominated the cotton effect around 250 nm. Thus, via comparing the ECD results with those of the experimental CD curves of **1** and **2**, the absolute configurations of C-3 and C-4 were determined to be (*R*), (*S*) and (*R*), (*R*) for **1** and **2**, respectively.

**Fig. 5:**
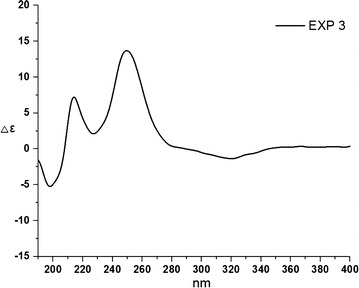
CD result of **3**

Compound **3** was obtained as yellow crystal. The molecular formula was determined to be C_11_H_12_O_4_ by HR–ESI–MS at *m/z* 231.0637 [M + Na]^+^. The ^1^H and ^13^C NMR spectral data of **3** were similar to those of **2**, expect for the disappearance of an acetoxy group at C-10. The absolute configuration of **3** was established by the analysis of its CD spectrum. A positive Cotton effect at 250 nm was shown in the CD spectrum (Fig. 5) of **3**, indicating the (3*R*,4*R*)-configuration same as **2**.

The cytotoxic activities of compounds 1-3 were evaluated using the HepG2, A549, LoVo and Hela cell lines, respectively. The IC_50_ values of these compounds were shown in Table 2. As a result, all the compounds exhibited moderate or less cytotoxic activity in vitro.

**Table 2:** IC_50_ value of the compounds **1**–**3** against four cell lines (μM)

Compound	Cell lines			
	HepG2	A549	LoVo	Hela
1	87.62	> 100	93.17	53.84
2	79.20	> 100	62.74	61.91
3	42.36	81.91	73.57	86.43

## Methods

### General experimental procedures

The UV spectrum was recorded on a Shimadzu UV-2201 spectrophotometer (Shimadzu Corporation, Kyoto, Japan). The IR spectrum was obtained from a Bruker IFS-55 spectrophotometer using a KBr pellet (Bruker Optik BmbH, Ettlingen, Germany). The HR-ESI–MS data were obtained on a microTOF-Q Bruker mass instrument (Bruker Daltonics, Billerica, MA, USA). CD spectra were recorded with a Biologic MOS-450 spectrometer using MeOH as solvent. 1D and 2D NMR spectra were run on a Bruker AVANCE 600 spectrometer (Bruker BioSpin, Rheinstetten, Germany). ^1^H chemical shifts (*δ*_H_) were measured in ppm, relative to TMS, and ^13^C chemical shifts (*δ*_C_) were measured relative to DMSO-*d*_6_ and converted to TMS scale. Column chromatography (CC) was performed on Silica gel (200–300 mesh; Qingdao Marine Chemical Co., Qingdao, China) and Sephadex LH-20 (Pharmacia, Uppsala, Sweden) columns. HPLC was performed on a Shimadzu LC-10AVP liquid chromatograph with a YMC-pack C18 (ODS) column (10 × 250 mm, 5 μm, apan) and a Shimadzu LC-8AVP liquid chromatograph with a Diamonsil C18 (ODS) column (4.6 × 250 mm, 5 μm, China). All reagents for isolation were HPLC or analytical grade and were purchased from Tianjin Damao Chemical Company (Tianjin, China). Fetal bovine serum and Dulbecco’s modified eagle medium (DMEM) were from Thermo Fisher Scientific, 96-well flat bottom plate were purchased from Corning Inc. (NY, USA), 3-[4,5-dimethyl-2-thiazolyl]-2,5 diphenyltetrazolium bromide (MTT) and dimethyl sulfoxide (DMSO) were purchased from Sigma-Aldrich Corporation (MA, USA).

## Materials

*Radix* Glycyrrhizae was purchased from Anhui Yishengyuan Traditional Chinese Medicine Pellets Co., Ltd., P. R. China, and all the materials were identified by Dr. Xiao Fu, Department of Traditional Chinese Medicine, First Affiliated Hospital of Jinzhou Medical University. The voucher specimen (20150610) has been deposited at First Affiliated Hospital of Jinzhou Medical University.

### Extraction and isolation

*Radix* Glycyrrhizae (25 kg) was cut and extracted with 70% EtOH for two times. The combined extracts were concentrated in vacuo to yield a residue, and the residue was then suspended in H_2_O and successively partitioned with petroleum ether, dichloromethane (CH_2_Cl_2_), ethyl acetate (EtOAc). The EtOAc crude extracts (2.3 kg) were applied on a silica gel column and eluted with petroleum ether-acetone gradient (from 500:0 to 0:100) to afford nine fractions. Fr. 6 was subjected to Sephadex LH-20, semi-preparative HPLC to yield compound **1** (12.0 mg) and **2** (9.2 mg). Fr. 7 was subjected to Sephadex LH-20, semi-preparative HPLC to yield compound **3** (15 mg).

#### ((3R,4S)-4,8-Dihydroxy-3-methyl-1-oxoisochroman-5-yl)methyl acetate (**1**)

Yellow needle crystal (CH_3_OH); UV (MeOH) *λ*_max_(log *ε*) 214, 315 nm; IR (KBr) *ν*_max_ 3408.9, 2920.2, 2850.0, 1674.3, 1446.1,1384.2, 1207.2, 1138.8 cm^−1^; CD (mdeg): Δ*ε*_212 nm_ + 25.0,Δ*ε*_250 nm_ − 14.6, Δ*ε*_290 nm_ + 5.6; ^1^H and ^13^C-NMR spectral data, see Table 1. HR-ESI–MS: *m/z* 289.0681 [M + Na]^+^ (calcd. for C_13_H_14_O_6_Na, 289.0683).

#### ((3R,4R)-4,8-Dihydroxy-3-methyl-1-oxoisochroman-5-yl)methyl acetate (**2**)

Yellow needle crystal (CH_3_OH); UV (MeOH) *λ*_max_(log *ε*) 218, 316 nm; IR (KBr) *ν*_max_ 3418.5, 2920.1, 2850.8, 1675.2, 1477.9,1383.7, 1208.1, 1171.9 cm^−1^; CD (mdeg): Δ*ε*_198 nm_ − 12.5,Δ*ε*_212 nm_ + 5.0,Δ*ε*_250 nm_ + 25.0, Δ*ε*_316 nm_ − 3.7;^1^H and ^13^C-NMR spectral data, see Table 1. HR–ESI–MS: *m/z* 289.0670 [M + Na]^+^ (calcd. for C_13_H_14_O_6_Na, 289.0683).

#### (3R,4R)-4,8-Dihydroxy-3,5-dimethylisochroman-1-one (**3**)

Yellow needle crystal (CH_3_OH); CD (mdeg): Δ*ε*_198 nm_ − 6.5,Δ*ε*_212 nm_ + 7.0,Δ*ε*_250 nm_ + 13.8, Δ*ε*_316 nm_ − 1.1;^1^H and ^13^C-NMR spectral data, see Table 1. HR-ESI-MS: *m/z* 231.0637 [M + Na]^+^ (calcd. for C_11_H_12_O_4_Na, 231.0628).

### Cytotoxic activity assay

Four cell lines including HepG2, A549, LoVo and Hela cell lines were purchased from the American Type Culture Collection. All the cell lines were used to evaluate the cytotoxic activities of compounds 1-3 in vitro by the method of MTT. Briefly, HepG2, A549, LoVo and Hela cells were seeded in 96-well flat bottom plates at a density of about 1 × 10^4^ cells/well, respectively. After incubating 12–18 h, 20 μL of compounds 1–3 were added into each well at a final concentration of 1, 5, 10, 25, 50, 100 and 200 μΜ. All the cells in the plates were incubated for another 48 h respectively. Subsequently, cell lines were incubated with MTT at the concentration of 0.5 mg/mL for 4 h, and then the cells were re-suspended in 150 μL of Dimethyl sulfoxide (DMSO). Inhibitory concentrations of compounds were calculated and half maximal inhibitory concentrations (IC_50_) values were confirmed. 5-Fluorouracil and dimethyl sulfoxide (DMSO, 0.1%, v/v) were used as positive control and negative control, respectively.

## Conclusion

A mount of chemical constituents have been isolated and identified from *Radix* Glycyrrhizae. Triterpenoids including glycyrrhizic acid, glycyrrhetinic acid and flavonoids including isoliquiritigenin, liquiritigenin, isoliquiritin, Licochalcone A, B and E are considered as the main characteristic constituents of the herb. And the anticancer bioactivities of these characteristic compounds were assayed frequently. Compared to triterpenoids, flavonoids possessed stronger anticancer bioactivities. In this study, three dihydroisocoumarins (**1**–**3**) showed the less toxicities on A549 and HepG2 cell lines than that of flavonoids constituents reported previously. IC_50_ value of isoliquiritigenin, licochalcone A and E on A549 cell lines were 18.5, 14.3 and 17.3 μM, respectively. Licochalcone A possessed almost the same toxicity on HepG2 cell lines (IC_50_, 10 μM). But the dihydroisocoumarins (**1**–**3**) showed more toxicity on A549 cell lines than that of Glycyrrhizic acid [7]. Besides anticancer activity, dihydroisocoumarin and its derivatives also exhibited anti-inflammatory and anti-bacterial effects [8, 9]. And these bioactivities assay were also the aim of our research in the future project.

## Additional file

**Additional file 1.** NMR and MS spectrum of compound **1**–**3**.
