# Supplementary material for: Dihydroisocoumarins from Radix Glycyrrhizae
Source: Chem Cent J. 2018 May 11;12:58. doi: 10.1186/s13065-018-0427-0 (PMC5945569; doi:10.1186/s13065-018-0427-0)
Supplement: Supplementary file 1 — Additional file 1. NMR and MS spectrum of compound 1–3. [file 13065_2018_427_MOESM1_ESM.docx]

NMR and MS spectrum of compound **1**-**3**.


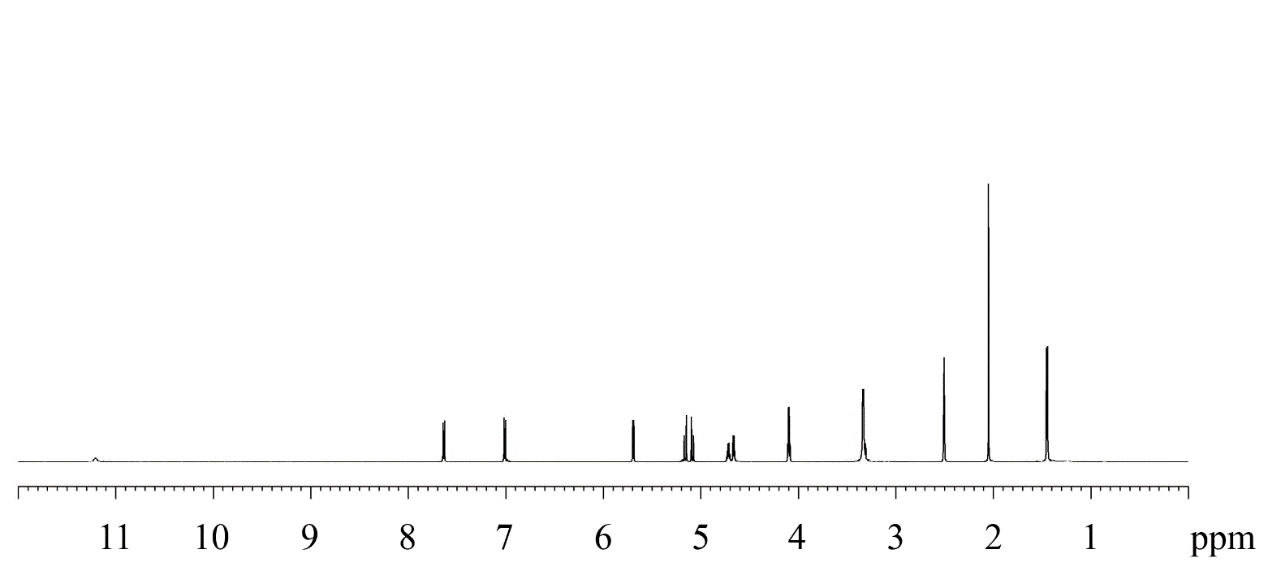


^1^H-NMR spectra of Compound **1**


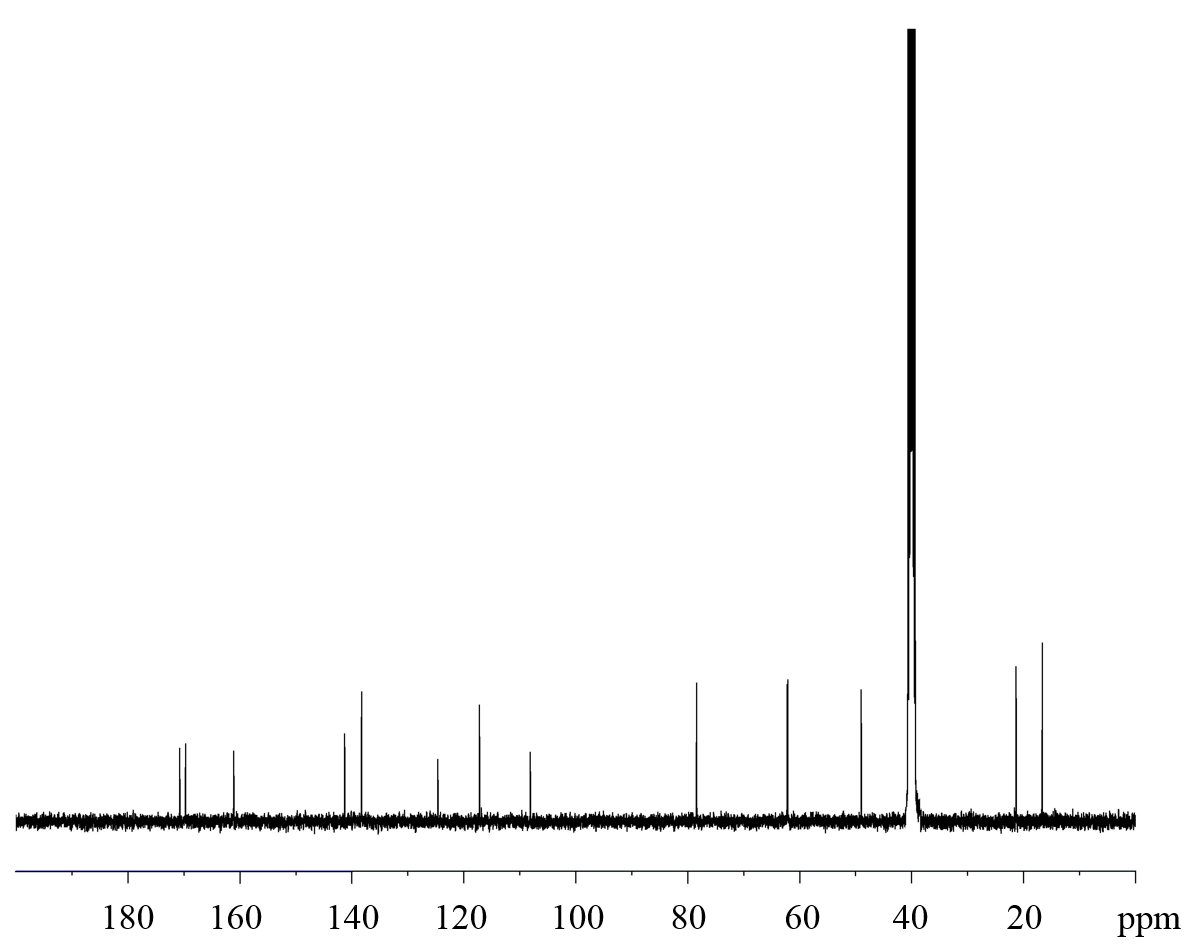


^13^C-NMR spectra of Compound **1**


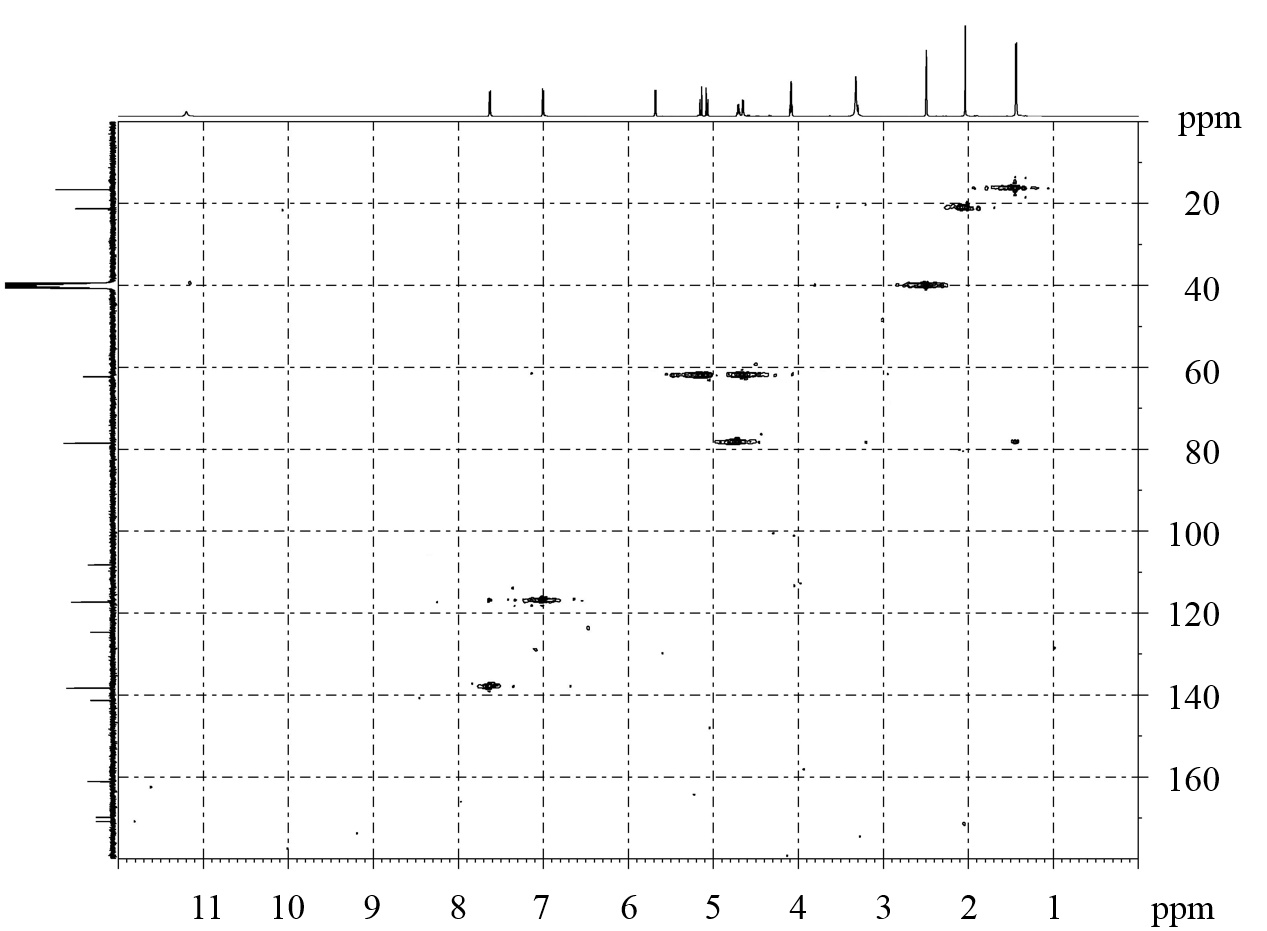


HSQC spectra of Compound **1**


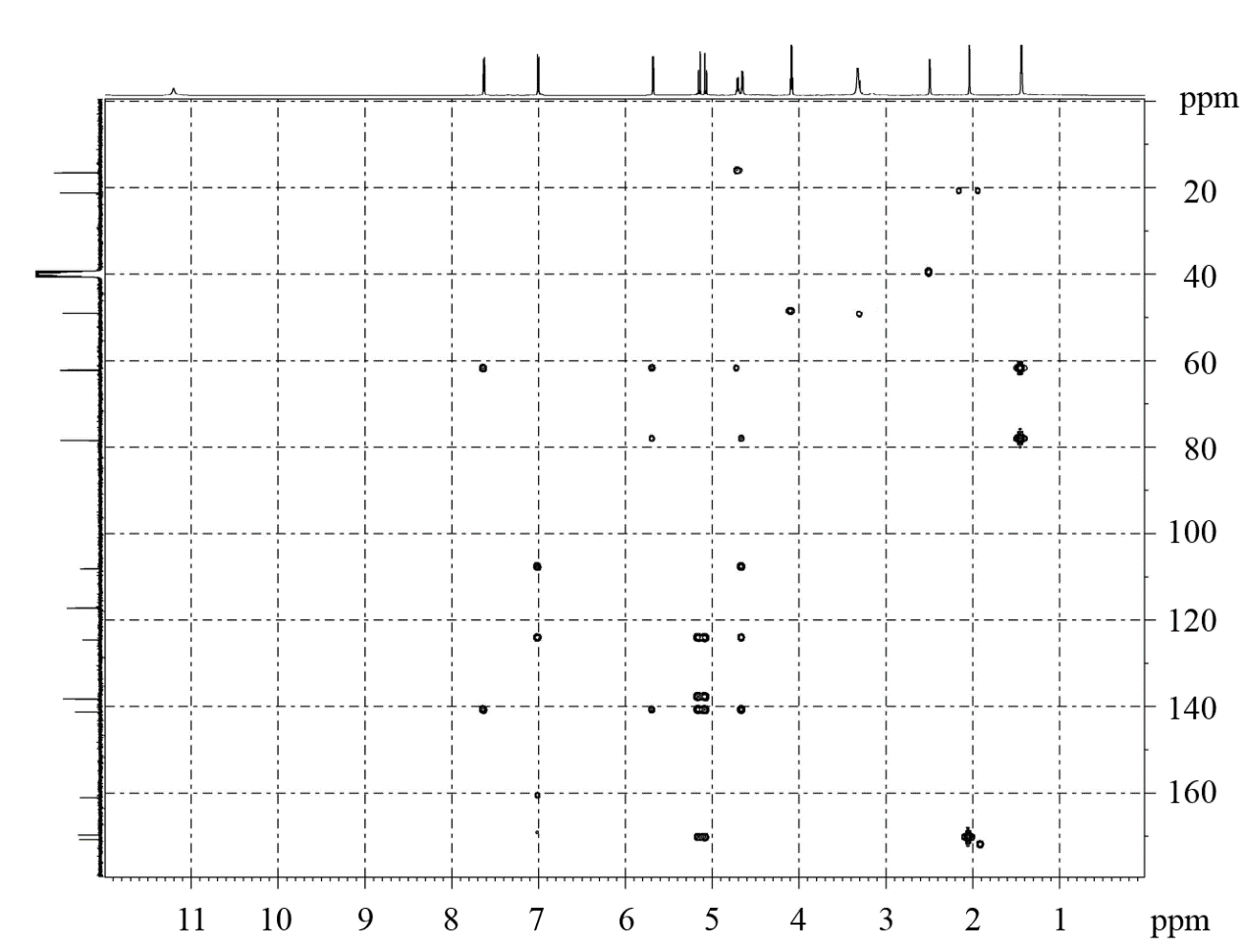


HMBC spectra of Compound **1**


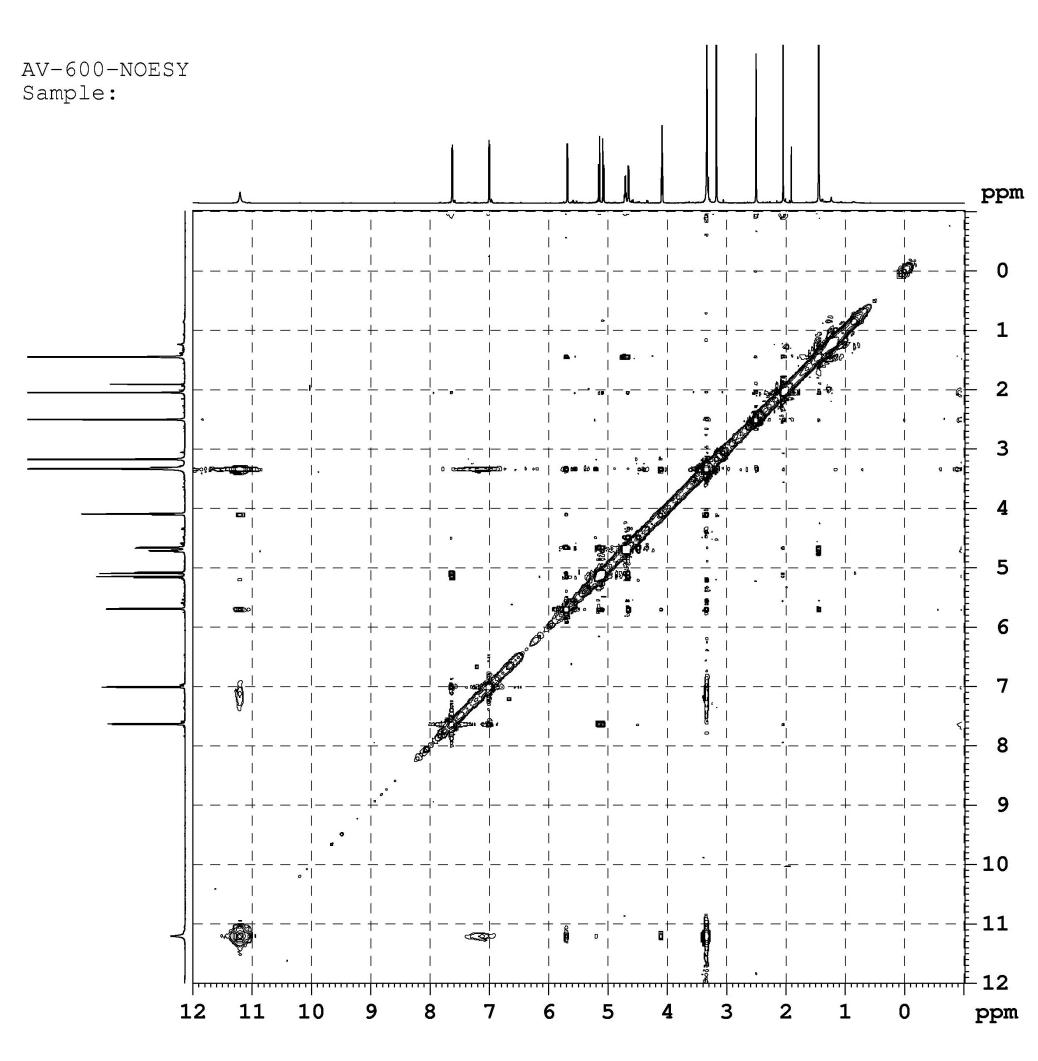


NOESY spectra of Compound **1**


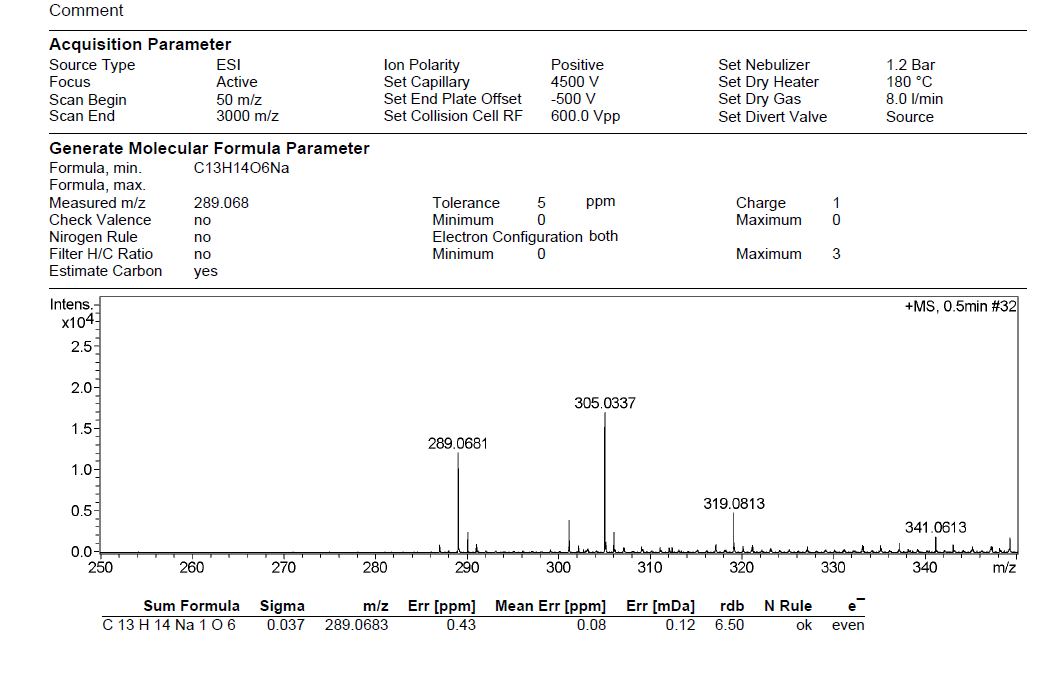


MS spectra of Compound **1**


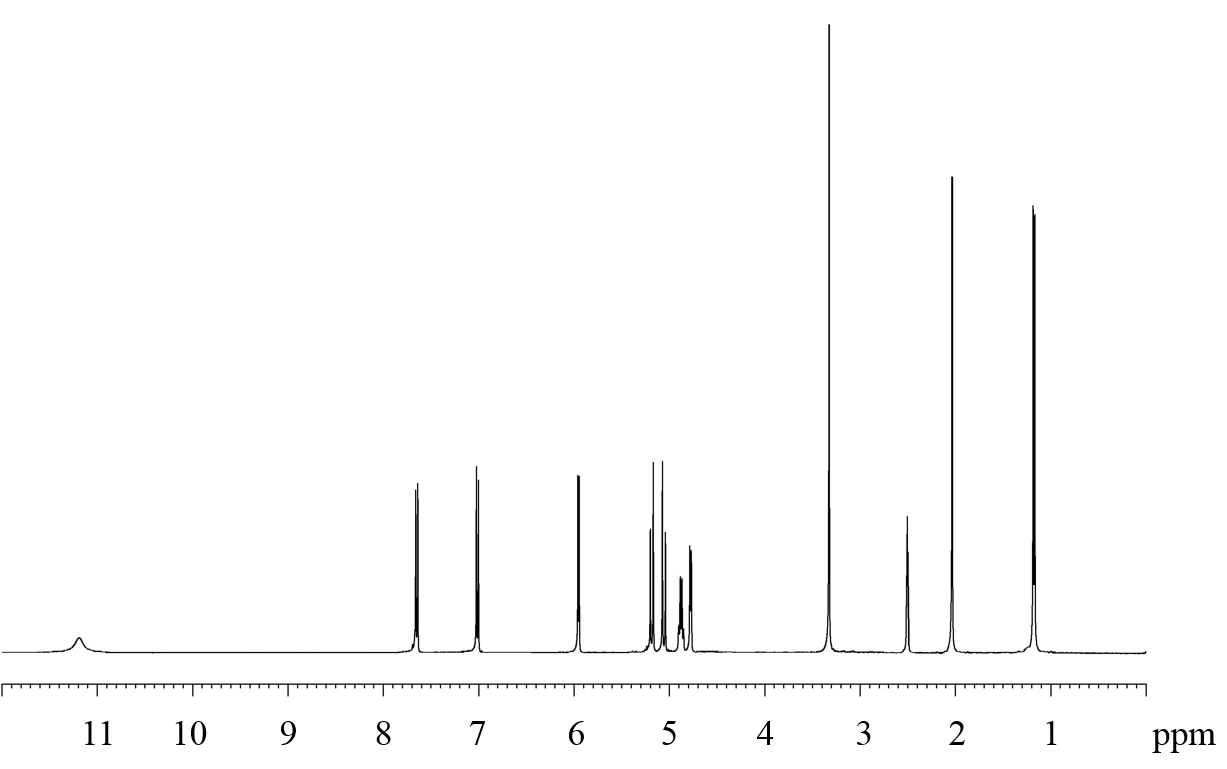


^1^H-NMR spectra of Compound **2**


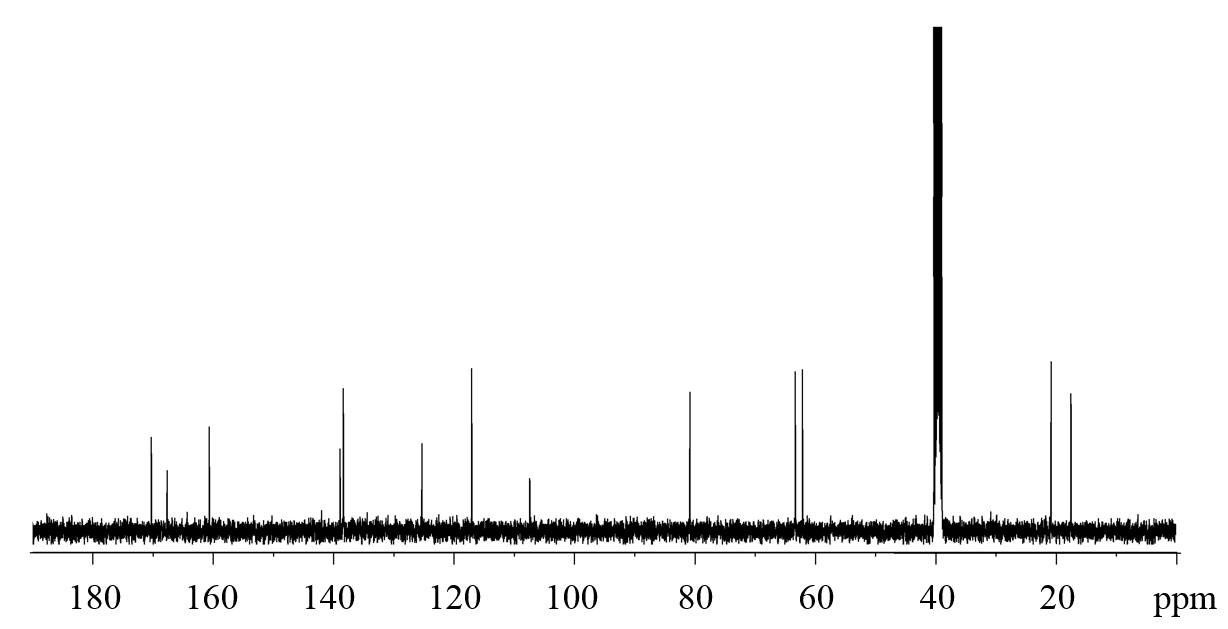


^13^C-NMR spectra of Compound **2**


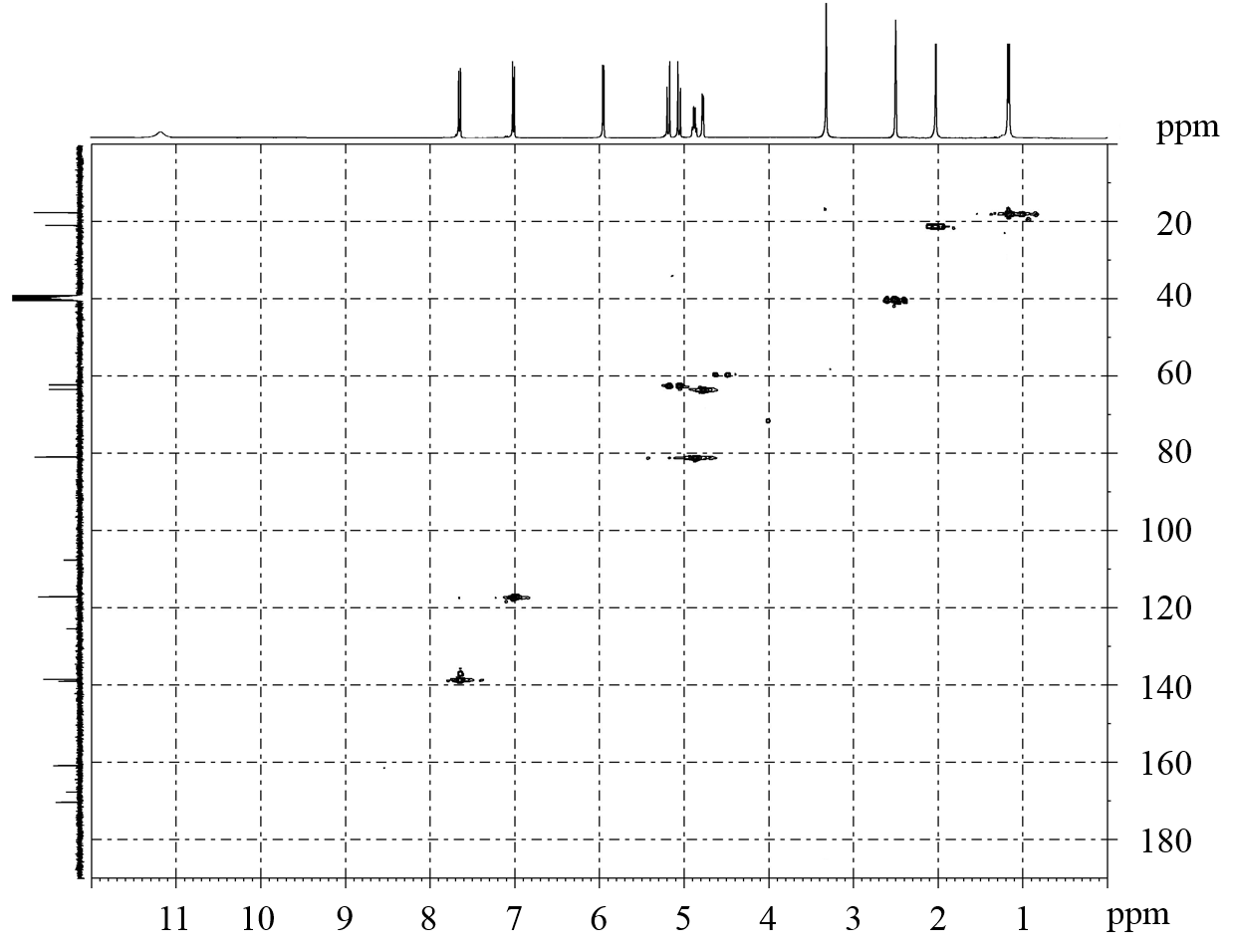


HSQC spectra of Compound **2**


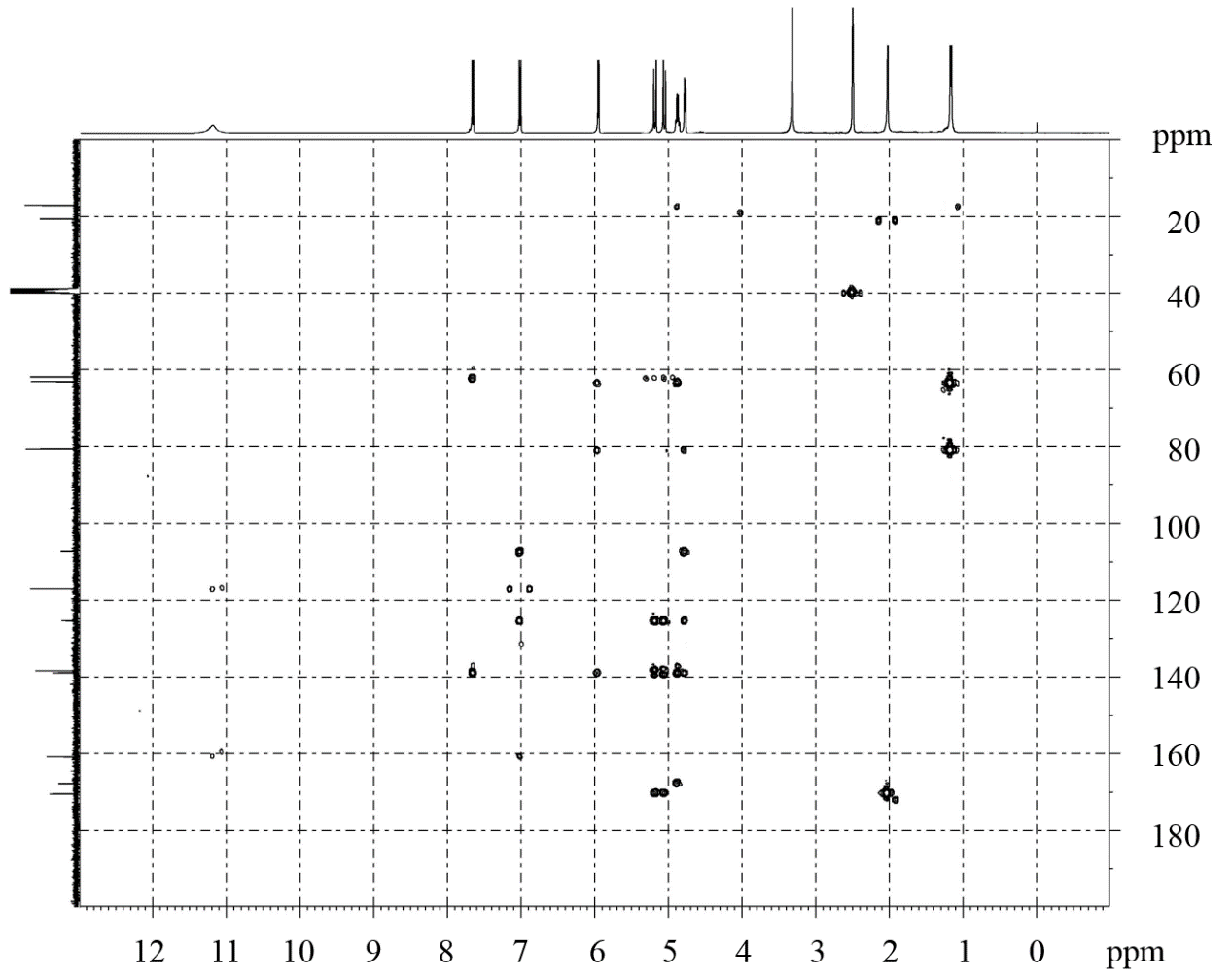


HMBC spectra of Compound **2**


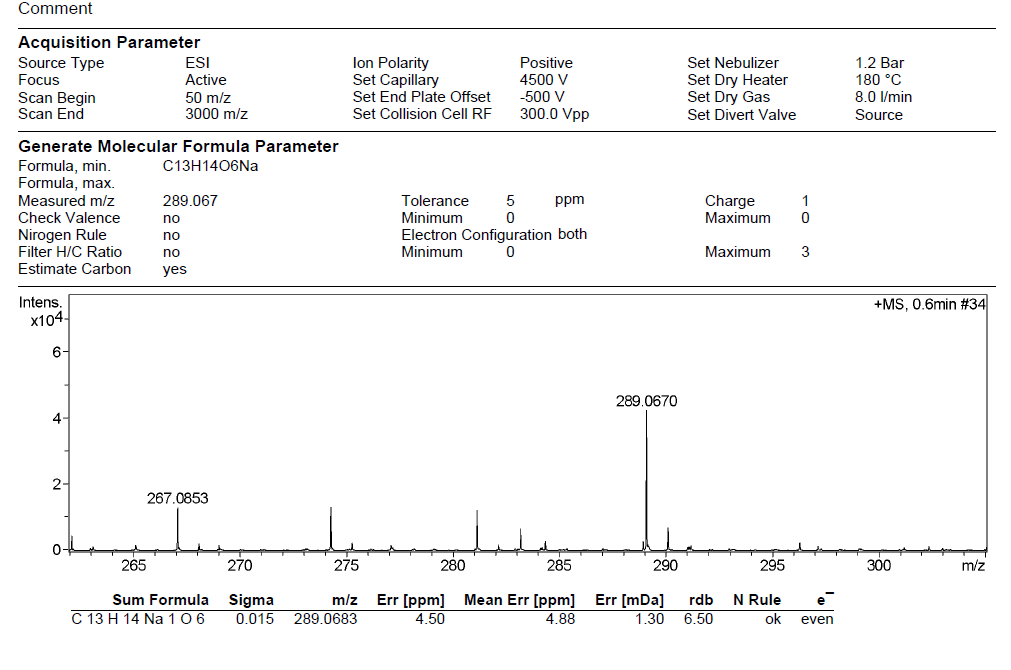


MS spectra of Compound **2**


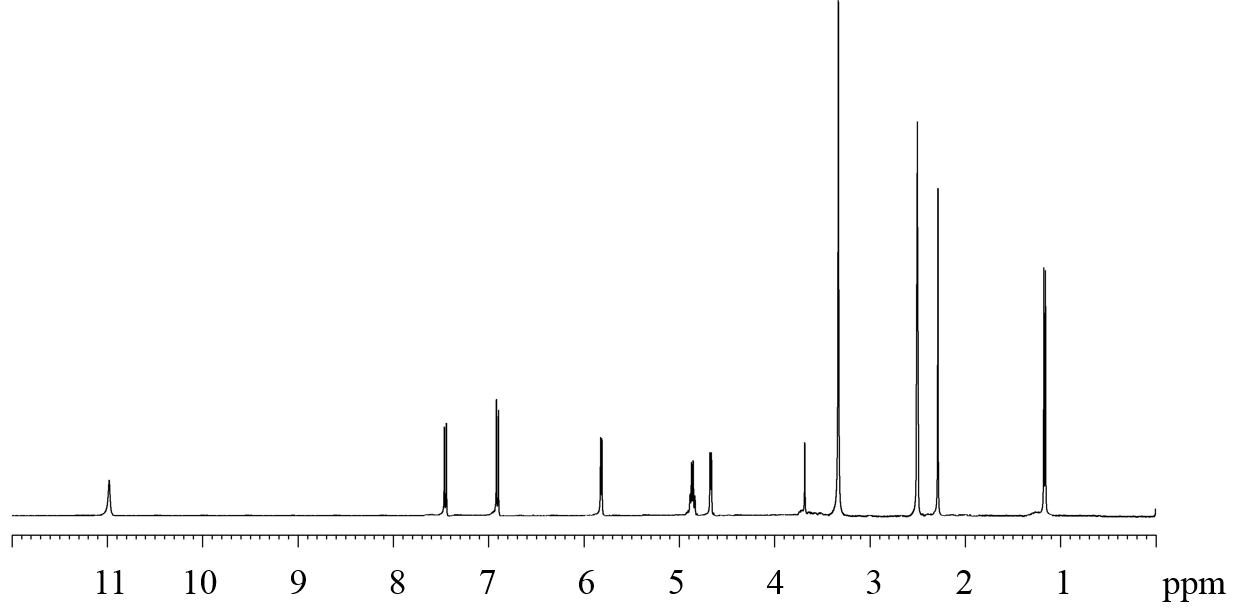


^1^H-NMR spectra of Compound **3**


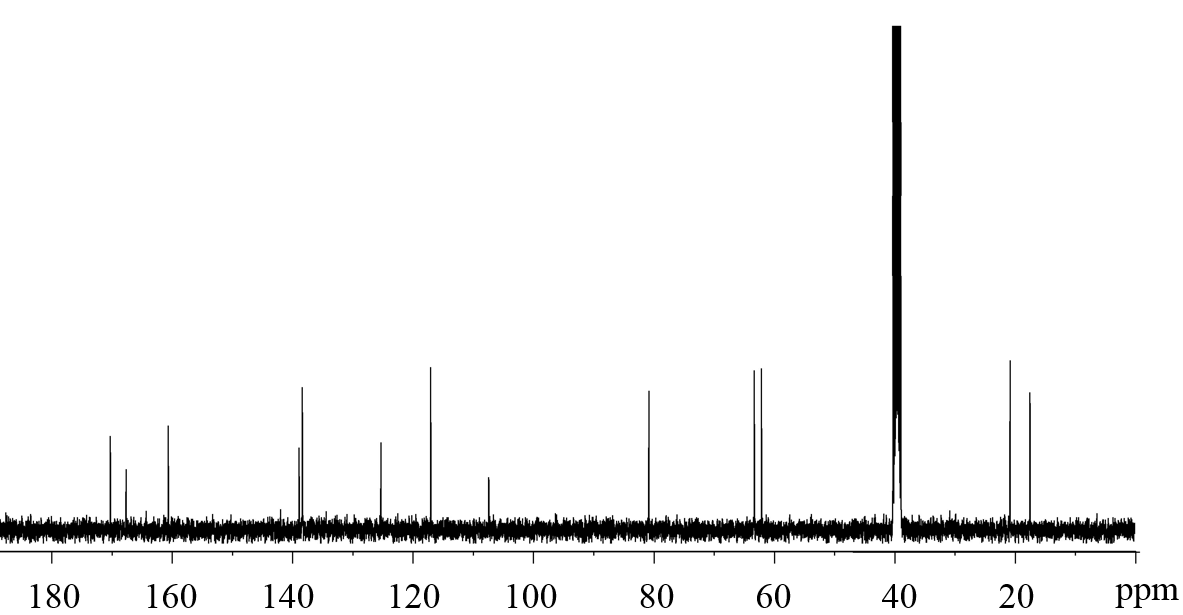


^13^C-NMR spectra of Compound **3**


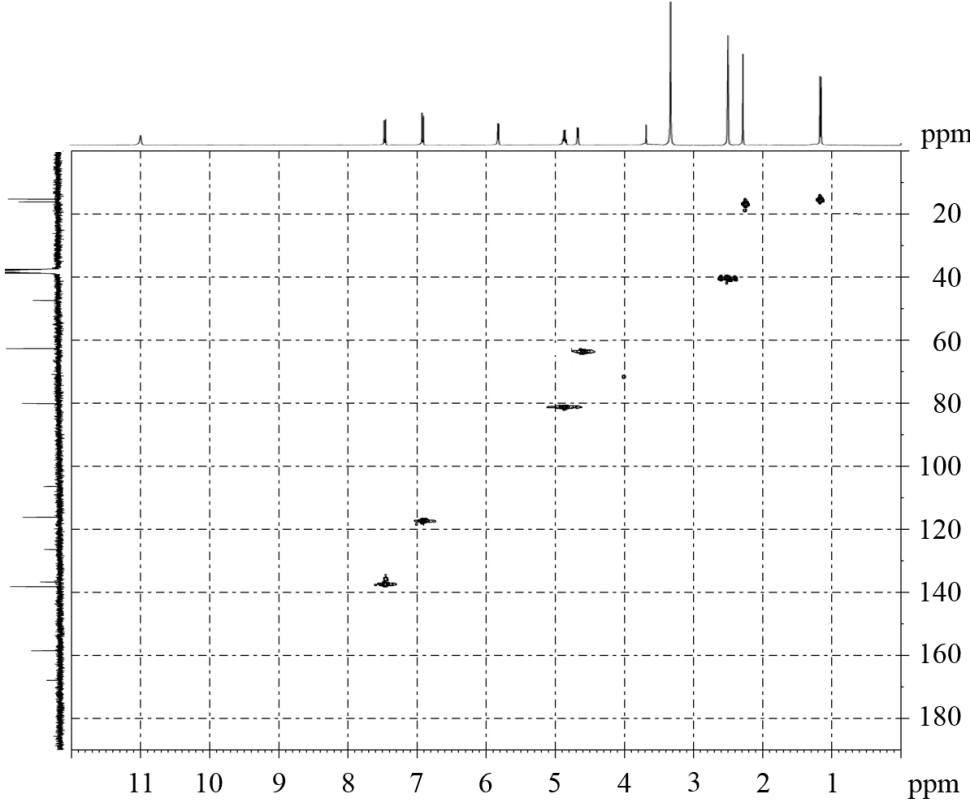


HSQC spectra of Compound **3**


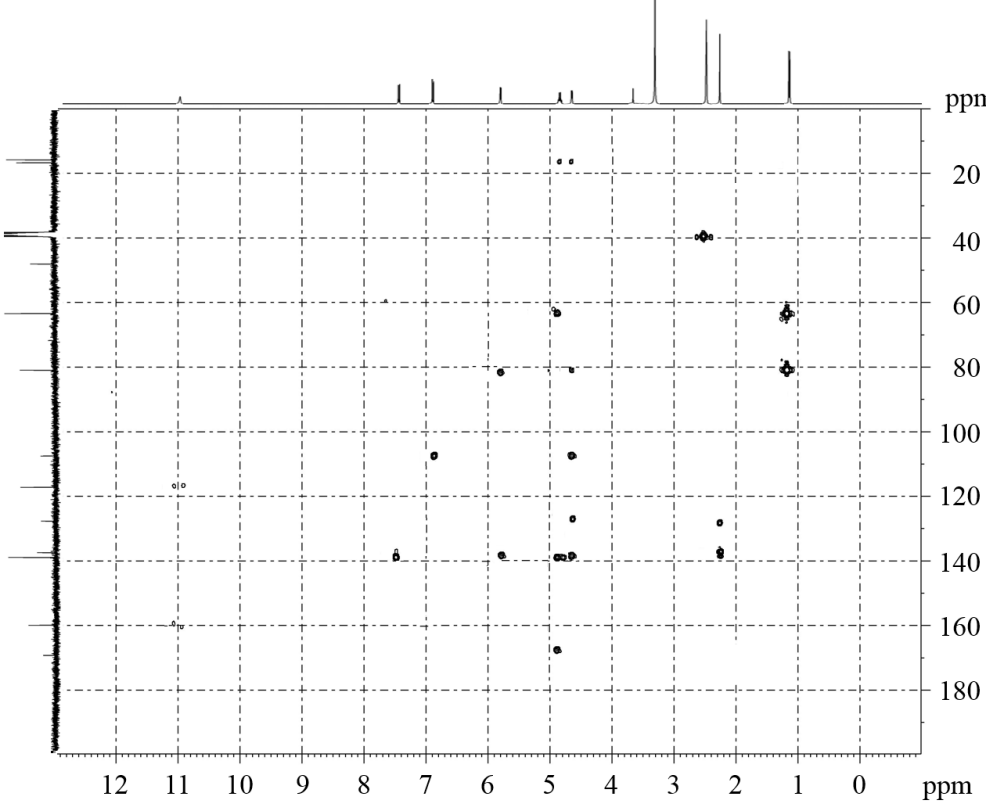


HMBC spectra of Compound **3**


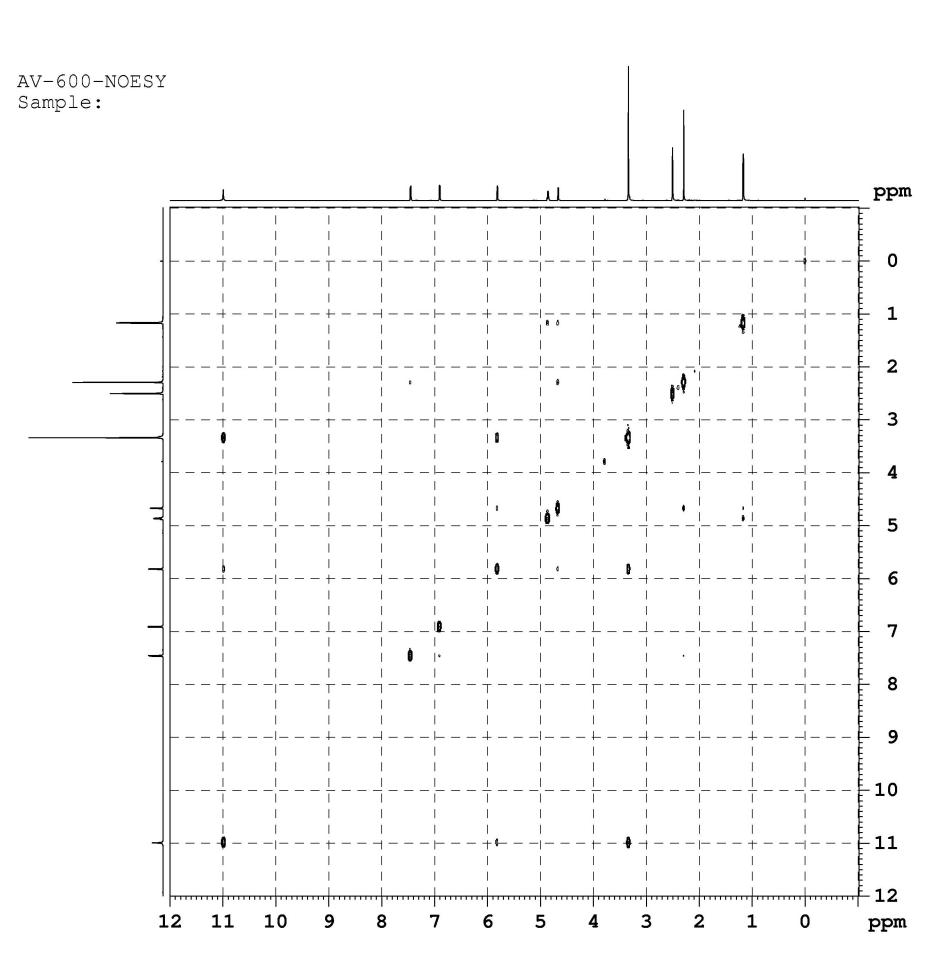


NOESY spectra of Compound **3**


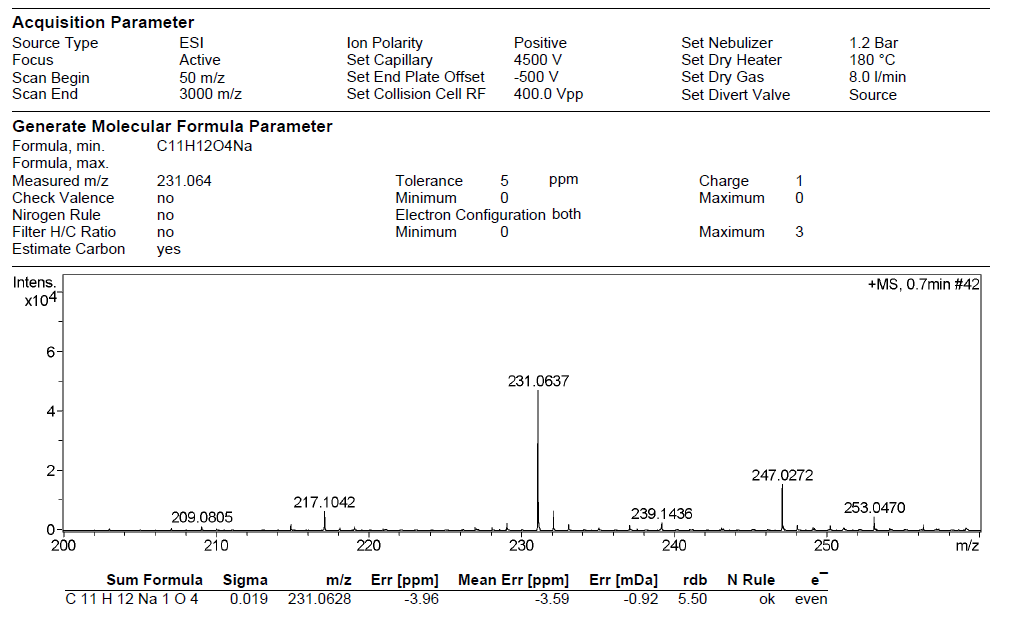


MS spectra of Compound **3**
